# A Study to Assess the Prevalence of Depression, Anxiety, and Stress and Their Association With Emotional Intelligence in Adult Males in Chengalpattu District

**DOI:** 10.7759/cureus.87893

**Published:** 2025-07-14

**Authors:** Nikhil C M, Shanthi Edward, Angeline Grace

**Affiliations:** 1 Community Medicine, Sree Balaji Medical College and Hospital, Chennai, IND

**Keywords:** angst, emotional depression, mental health, social intelligence, stress disorder

## Abstract

Background and aim

The most common mental health problems worldwide are depression, anxiety, and stress, and the emotional expression of the same is discouraged among men due to societal expectations, increasing the morbidity and mortality caused by these conditions. People with high emotional intelligence have a better understanding of their own and others' emotions, helping them to better solve their problems and manage stress, which effectively reduces their stress and anxiety. This study aimed to assess the prevalence of depression, anxiety, and stress and their association with emotional intelligence in adult males in the Chengalpattu district.

Methodology

A cross-sectional study was done among the adult males (over 18 years of age) in the field practice area of the Urban Health Training Centre of a tertiary medical college in the Chengalpattu district, Tamil Nadu. A total of 337 people had participated in the study. A pretested semi-structured questionnaire was used to collect sociodemographic details and information on factors associated with depression, anxiety, and stress. The Schutte Self-Report Emotional Intelligence Test (SSEIT) was used to assess the emotional intelligence of the individuals. Data analysis was done using SPSS software version 25 (Armonk, NY: IBM Corp.).

Results

The prevalence of depression, anxiety, and stress was found to be 27.3%, 43.6%, and 21.7%, respectively. A total of 24.3% of people had low emotional intelligence. Having low emotional intelligence was found to be significantly associated with depression, anxiety, and stress. Having less perceived social support, adverse childhood experiences, and alcohol and tobacco consumption were also found to be strongly associated with depression, anxiety, and stress.

Conclusion

Emotional intelligence is inversely associated with levels of depression, anxiety, and stress among the adult male population, underscoring the protective role of emotional intelligence in mental well-being. Future interventions aiming to reduce the burden of common mental disorders should consider fostering emotional intelligence as a core component.

## Introduction

Depression, anxiety, and stress are among the most common mental health problems worldwide. Among men, the emotional expression of any of these conditions is discouraged and under-recognized because of the societal expectations put upon them by their family and peers [1]. When compared with women, men are less likely to talk about these problems or ask for help, increasing the chances of these conditions becoming underreported and increasing the morbidity and mortality caused by these mental health conditions [2]. In India, depression among men is one of the major concerns of public health due to the under-recognition of these conditions because of the impact of cultural norms and stigma faced by men who seek mental health support [3]. Anxiety, on the other hand, if left unaddressed, can lead to depression [4]. Challenges faced in family life, such as financial stress and stress in the workplace, can lead to substance abuse, which, in turn, can lead to depression and anxiety [1].

Emotional intelligence (EI), which can be defined as an individual’s ability to effectively understand, perceive, and manage their emotions, has been found to play an important role in understanding mental health, especially among men, due to the various sociocultural challenges they face in their lifetime [5]. It has been found that individuals with high emotional intelligence have a better understanding of the emotions of themselves and others, which empowers them to effectively manage their relationships and provide better social support to others [6]. It also provides them with good problem-solving skills and stress management by providing them with better resilience to manage their day-to-day activities, which in turn effectively reduces their stress and anxiety [7].

Emotional intelligence plays an important role in managing family life, especially among men, who often sail across the traditional gender roles faced with them, which limits their emotional expression [8]. It has also been found that low EI among men, leading to conflicts within the family, especially between husband and wife, plays an important role in shaping the EI of their children, contributing to their mental health in their adult life. Children learn to express their feelings and manage conflicts, which in turn has a direct relationship with the EI of their parents [9].

In the workplace context, good EI can help in better management of workplace challenges and help them to effectively communicate, leading to better relationships with peers. This, in turn, could lead to a healthy work environment and promote mental health and well-being [10].

There is limited availability of studies exploring the association between depression, anxiety, and stress and emotional intelligence, particularly in the Indian context, where there is a high prevalence of mental health stigma and stunted emotional expression among men [11]. With this background, the present study was carried out among adult males residing in Chengalpattu district, with an objective to estimate the prevalence of depression, anxiety, and stress and to find its association with emotional intelligence.

## Materials and methods

Study setting

A cross-sectional study was done among the adult males (over 18 years of age) in the field practice area of the Urban Health Training Centre of a tertiary medical college in Chengalpattu district, Tamil Nadu. The study was done from April 2025 to May 2025.

Sample size calculation

In a meta-analysis done by Salve et al., the prevalence of depression ranged from 10% to 67% [12]. So the upper limit of 67% was taken as prevalence (P).

$n = \frac{Z^2 \cdot P \cdot Q}{L^2}$

Here, Z=1.96, 95% confidence interval, P=67%, Q=33%, and absolute precision L=5%, and by applying the formula above, the minimum required sample size was calculated to be 337.

Sampling method

The total adult population of Anakaputhur, urban field practice area of Sree Balaji Medical College and Hospital, is 15,403, who are distributed among four wards, with a population of ward one being 3590, ward two being 3909, ward three being 3927, and ward four being 3978. The sample size required for our study is 337, which was proportionately distributed among four wards using probability-proportional-to-size sampling (PPS sampling). Applying PPS sampling, the required sample size was 78 for ward one, 85 for ward two, 86 for ward three, and 88 for ward four. To achieve this, a door-to-door survey was conducted in each ward, starting from the first house on the street at the centre of the ward, until the required sample size was achieved.

Study tools

A pre-tested semi-structured questionnaire was used to collect sociodemographic details. The pre-validated Depression, Anxiety and Stress Scale-21 (DASS-21) questionnaire was used to assess depression, anxiety, and stress. It is a 21-item questionnaire that consists of three subscales to assess depression, anxiety, and stress, respectively. It uses a four-point Likert scale ranging from "0" (does not apply to me) to "3" (applies to me most of the time) to rate the participants’ experiences. The summed-up scores in each subscale are used to categorize their condition into mild, moderate, severe, and extreme [13]. DASS-21 is a validated scale to measure depression, anxiety, and stress in the adult population [14]. The validated Schutte Self-Report Emotional Intelligence Test (SSEIT) was used to assess the emotional intelligence of the individuals. It is a 33-item questionnaire using a five-point Likert scale for responses, ranging from "1" (strongly agree) to "5" (strongly disagree). A person with a summed-up score of less than 111 is considered to have low EI [15].

Data analysis

Data were entered into Microsoft Excel (Redmond, WA: Microsoft Corp.), and statistical analysis was performed using SPSS version 25 (Armonk, NY: IBM Corp.). Descriptive statistics are presented as tables. Bivariate analysis was performed using the chi-square test, and variables that were found to be statistically significant at the 95% CI were included in the logistic regression model. The strength of the association between depression, anxiety, and stress and their related factors was quantified using adjusted odds ratios.

## Results

Table 1 shows the sociodemographic features of the study participants and the study variables. The majority of the participants (189 {56.1%}) were aged more than 40 years. People belonging to the middle/lower class (255 {75.7%}) constituted a major chunk of the population. A total of 225 (66.8%) participants reported that they didn’t have any trauma/adverse childhood experiences. In total, 177 (52.5%) participants reported that they have people whom they can rely/on. A total of 135 (40.1%) study participants reported that they had less than 6 hours of sleep. Lastly, 152 (45.1%) participants said that they have consumed alcohol/tobacco.

**Table 1:** Frequency distribution of the study variables among the study participants.

S. no.	Variables	Category	Frequency, n (%)
1	Age	Less than 40 years	148 (43.9)
		More than 40 years	189 (56.1)
2	Alcohol/tobacco consumption	Yes	152 (45.1)
		No	185 (54.9)
3	Sleep	>6 hours	202 (59.9)
		<6 hours	135 (40.1)
4	Perceived social support	I have people whom I can rely/depend on	177 (52.5)
		I don’t have people whom I can rely/depend on	160 (47.5)
5	Trauma/adverse childhood experiences	Yes	112 (33.2)
		No	225 (66.8)
6	Socioeconomic status	Upper middle/upper class	82 (24.3)
		Middle/lower middle/lower class	255 (75.7)

The prevalence of depression, anxiety, stress, and low emotional intelligence is given in Table 2. Ninety-two (27.3%) participants had depression. Of the total sample population, 147 (43.6%) had anxiety. People with stress were low in number when compared to people with depression, anxiety, and low intelligence. Seventy-three (21.7%) participants had stress. It was estimated that 82 (24.3%) participants had low emotional intelligence.

**Table 2:** Frequency distribution of prevalence of depression, anxiety, stress, and emotional intelligence.

S. no.	Variables	Category	Frequency, n (%)
1.	Depression	Has depression	92 (27.3)
		Doesn’t have depression	245 (72.7)
2.	Anxiety	Has anxiety	147 (43.6)
		Doesn’t have anxiety	190 (56.4)
3.	Stress	Has stress	73 (21.7)
		Doesn’t have stress	264 (78.3)
4.	Emotional intelligence	Low emotional intelligence	82 (24.3)
		Normal emotional intelligence	255 (75.7)

The bivariate analysis and logistic regression between depression and its related variables are given in Table 3. Multivariate logistic regression analysis showed that participants aged <40 years were significantly more likely to report depression compared to those aged >40 years (AOR=4.561, 95% CI: 2.734-7.312). Sleep duration less than 6 hours was also significantly associated with higher odds of depression (AOR=4.275, 95% CI: 2.681-6.943). Participants reporting inadequate perceived social support had a significantly higher likelihood of depression compared to those lacking such support (AOR=3.921, 95% CI: 2.148-7.426). Those with a history of trauma or adverse childhood experiences were more likely to experience depression (AOR=6.215, 95% CI: 4.135-10.058). Notably, low emotional intelligence emerged as a strong predictor of depression (AOR=9.853, 95% CI: 5.549-16.679). Socioeconomic status and alcohol and tobacco consumption were not significantly associated with depression in the adjusted model.

**Table 3:** Bivariate analysis and logistic regression between depression and its related variables.

S. no.	Variables	Depression		Odds ratio	Unadjusted OR 95% CI	Adjusted odds ratio	Adjusted OR 95% CI
		Yes, n (%)	No, n (%)				
1	Age of the study participant						
	<40 years	68 (45.9)	80 (54.1)	5.844	3.417-9.993	4.561	2.734-7.312
	>40 years	24 (12.7)	165 (87.3)				
2	Alcohol/tobacco consumption						
	Yes	37 (24.3)	115 (75.7)	0.760	0.468-1.237	-	-
	No	55 (29.7)	130 (70.3)				
3	Sleep						
	<6 hours	72 (35.6)	130 (64.4)	3.185	1.828-5.549	4.275	2.681-6.943
	>6 hours	20 (14.8)	115 (85.2)				
4	Perceived social support						
	I don’t have people whom I can rely/depend on	72 (40.7)	105 (59.3)	4.800	2.752-8.372	3.921	2.148-7.426
	I have people whom I can rely/depend on	20 (12.5)	140 (87.5)				
5	Trauma/adverse childhood experiences						
	Yes	57 (50.9)	55 (49.1)	5.626	3.355-9.434	6.215	4.135-10.058
	No	35 (15.6)	190 (84.4)				
6	Socioeconomic status						
	Upper middle/upper class	32 (39)	50 (61)	2.080	1.225-3.533	1.713	0.845-3.124
	Middle/lower middle/lower class	60 (23.5)	195 (76.5)				
7	Emotional intelligence						
	Low emotional intelligence	52 (63.4)	30 (36.6)	9.317	5.311-16.343	9.853	5.549-16.679
	Normal emotional intelligence	40 (15.7)	215 (84.3)				

The results of bivariate analysis and logistic regression between anxiety and its related variables are given in Table 4. In multivariate analysis, participants aged <40 years were significantly more likely to experience anxiety compared to those aged >40 years (AOR=7.914, 95% CI: 3.857-13.546). Those who reported not having people to rely on exhibited a higher likelihood of anxiety (AOR=4.761, 95% CI: 2.943-7.128). Trauma or adverse childhood experiences were significantly associated with anxiety (AOR=15.875, 95% CI: 10.279-26.481). Low emotional intelligence also showed a significant association with anxiety (AOR=3.574, 95% CI: 2.283-5.368). While alcohol/tobacco consumption, sleep duration, and socioeconomic status were associated with anxiety in unadjusted analyses, these associations lost statistical significance in the multivariate model.

**Table 4:** Bivariate analysis and logistic regression between anxiety and its related variables.

S. no	Variables	Anxiety		Odds ratio	Unadjusted OR 95% CI	Adjusted odds ratio	Adjusted OR 95% CI
		Yes, n (%)	No, n (%)				
1	Age of the study participant						
	<40 years	108 (73)	40 (27)	10.385	6.263-17.219	7.914	3.857-13.546
	>40 years	39 (20.6)	150 (79.4)				
2	Alcohol/tobacco consumption						
	Yes	77 (50.7)	75 (49.3)	1.687	1.092-2.606	0.943	0.671-1.835
	No	70 (37.8)	115 (62.2)				
3	Sleep						
	<6 hours	107 (53)	95 (47)	2.675	1.686-4.243	1.482	0.843-2.573
	>6 hours	40 (29.6)	95 (70.4)				
4	Perceived social support						
	I don’t have people whom I can rely/depend on	112 (63.3)	65 (36.7)	6.154	3.794-9.981	4.761	2.943-7.128
	I have people whom I can rely/depend on	35 (21.9)	125 (78.1)				
5	Trauma/adverse childhood experiences						
	Yes	92 (82.1)	20 (17.9)	14.218	8.031-25.172	15.875	10.279-26.481
	No	55 (24.4)	170 (75.6)				
6	Socioeconomic status						
	Upper middle/upper class	52 (63.4)	30 (36.6)	2.919	1.742-4.891	1.726	0.845-3.567
	Middle/lower middle/lower class	95 (37.3)	160 (62.7)				
7	Emotional intelligence						
	Low emotional intelligence	52 (63.4)	30 (36.6)	2.919	1.742-4.891	3.574	2.283-5.368
	Normal emotional intelligence	95 (37.3)	160 (62.7)				

Table 5 shows the results of bivariate analysis and logistic regression between stress and its related variables. Participants aged <40 years had significantly higher odds of reporting stress compared to those aged >40 years (AOR=9.357, 95% CI: 3.815-27.423). Alcohol or tobacco use was significantly associated with stress (AOR=4.152, 95% CI: 2.142-9.415). Individuals who slept less than six hours were more likely to report stress (AOR=3.814, 95% CI: 2.751-4.827). Similarly, lack of perceived social support was significantly associated with stress (AOR=3.428, 95% CI: 1.752-6.184). The presence of trauma or adverse childhood experiences was a strong predictor of stress (AOR=11.276, 95% CI: 5.815-22.423). Emotional intelligence was again significantly associated; those with low emotional intelligence had a markedly higher likelihood of experiencing stress (AOR=4.591, 95% CI: 3.148-6.957). Socioeconomic status did not show a statistically significant association with stress in the multivariate model.

**Table 5:** Bivariate analysis and logistic regression between stress and its related variables.

S. no.	Variables	Stress		Odds ratio	Unadjusted OR 95% CI	Adjusted odds ratio	Adjusted OR 95% CI
		Yes, n (%)	No, n (%)				
1	Age of the study participant						
	<40 years	64 (43.2)	84 (56.8)	15.238	7.239-32.074	9.357	3.815-27.423
	>40 years	9 (4.8)	180 (95.2)				
2	Alcohol/tobacco consumption						
	Yes	57 (37.5)	95 (62.5)	6.338	3.448-11.650	4.152	2.142-9.415
	No	16 (8.6)	169 (91.4)				
3	Sleep						
	<6 hours	93	109	2.259	1.414-3.610	3.814	2.751-4.827
	>6 hours	37	98				
4	Perceived social support						
	I don’t have people whom I can rely/depend on	57 (32.2)	120 (67.8)	4.275	2.334-7.830	3.428	1.752-6.184
	I have people whom I can rely/depend on	16 (10)	144 (90)				
5	Trauma/adverse childhood experiences						
	Yes	57 (50.9)	55 (49.1)	13.538	7.217-25.394	11.276	5.815-22.423
	No	16 (7.1)	209 (92.9)				
6	Socioeconomic status						
	Upper middle/upper class	17 (20.7)	65 (79.3)	0.929	0.505-1.712		
	Middle/lower middle/lower class	56 (22)	199 (78)				
7	Emotional intelligence						
	Low emotional intelligence	33 (40.2)	49 (59.8)	3.620	2.077-6.310	4.591	3.148-6.957
	Normal emotional intelligence	40 (15.7)	215 (84.3)				

## Discussion

In the present study, the prevalence of depression was 27.2%. In a study by Gandhi and Kishore, the prevalence was 40% among software professionals in Delhi [16]. According to the National Mental Health Survey, its prevalence is 5.25% [17]. Similarly, lower prevalence was observed in a study by Deswal and Pawar in Pune (3.14%) and Rao et al. in a rural area of South India (6.62%) [18,19]. The prevalence of anxiety disorder (AD) was 43.6%, and almost half of the participants were diagnosed with anxiety. According to the National Mental Health Survey, the prevalence of AD is 2.59% [20]. A meta-analysis conducted by Reddy and Chandrashekar found a pooled prevalence of 20.7% [21]. Stress was present in 21.6% of participants. In a study by Sahoo and Khess, the prevalence of stress was 20.2% [22]. Comparatively lower prevalence (5%) was reported in a study conducted by Srinivasan et al. in Puducherry [23]. This variation in the findings may be due to the fact that depression and sociocultural factors influencing it may have varied between the study areas.

We observed that emotional intelligence was significantly associated with depression, anxiety, and stress. In a study by Grases et al., emotional intelligence in the form of empathy and emotional competence was found to have a significant relationship with depression [24]. A similar observation was documented in meta-analyses by Schutte et al. [25] and Martins et al. [26]. These findings highlight the fact that people with depression should work to improve their interpersonal emotional skills, which could help in alleviating the morbidity associated with depression, as documented in a study by Grases et al. [24].

Regarding anxiety and emotional intelligence, a study conducted by Baudry et al. found that emotional intelligence was negatively associated with anxiety, meaning that higher emotional intelligence helps individuals regulate their emotions with more flexible and adaptive strategies that help them manage stressful situations and reduce their anxiety [27]. Even when faced with a situation in which they cannot change or modify the situation, those with high EI tend to manage the situation by reappraising and accepting it [28].

In a study done by Singh and Sharma, it was observed that high levels of emotional intelligence were inversely associated with perceived levels of acute and chronic stress [29]. Similar findings were observed in studies done by Landa et al. and Nikolaou and Tsaousis [30,31]. These findings relate to the fact that solving interpersonal problems by understanding the feelings of others helps people to lead a satisfied life, which in turn could help in reducing their stress levels [29].

Various other factors like adverse events in childhood, sleep less than 6 hours, and perceived social support were found to have a statistically significant association with depression, anxiety, and stress. A study done by Molnar et al. found a statistically significant association between childhood trauma and depression [32]. A study done by Kisely et al. found that childhood trauma, especially emotional abuse, was found to have a strong association with psychiatric morbidity, especially anxiety and depression [33]. This, in turn, plays a significant role in shaping the emotional intelligence of an individual, since it affects the brain areas that support emotional intelligence [34]. In a study done by Grey et al., there was a significant association between social support and depression, which in turn leads to poor sleep quality [35]. A study done by Dour et al. found that perceived support could have a mediation effect on depression and anxiety symptoms over time [36]. Therefore, measures at the family level, to increase social support, could in turn lead to a reduction in the psychiatric morbidities in the individual, such as depression and anxiety.

Strengths and limitations

The strengths of the study are that it addresses an underexplored area by examining the association between emotional intelligence and common mental disorders in adult males, a population often overlooked in mental health research, and the use of validated tools (DASS-21 and SSEIT) enhances the reliability of the findings. This study has certain limitations. Its cross-sectional design prevents inference of causality between emotional intelligence and mental health outcomes. Responses to DASS-21 and SSEIT scales may be subject to recall and social desirability biases, especially among men, due to cultural stigma.

## Conclusions

This study highlights a significant inverse relationship between emotional intelligence and levels of depression, anxiety, and stress among the adult male population. These findings underscore the protective role of emotional intelligence in mental well-being and support the integration of emotional intelligence training into mental health promotion strategies. Future interventions aiming to reduce the burden of common mental disorders should consider fostering emotional intelligence as a core component, particularly in high-stress environments and vulnerable populations.

## References

[1] (2025). Men and mental health: what are we missing?. https://www.aamc.org/news/men-and-mental-health-what-are-we-missing.

[2] (2025). Anxiety and depression in men. https://www.betterhealth.vic.gov.au/health/conditionsandtreatments/anxiety-and-depression-in-men.

[3] Mor M, Jayaseelan V, Kattimani S, Thyagaraju C, Kubera NS, Duraiswamy M (2024). Depression, anxiety, stress, and coping among men with infertility seeking treatment at a tertiary care hospital in South India: a mixed-method study. Indian J Psychiatry.

[4] Fisher K, Seidler ZE, King K, Oliffe JL, Robertson S, Rice SM (2022). Men's anxiety, why it matters, and what is needed to limit its risk for male suicide. Discov Psychol.

[5] Alam KK (20231). Emotional intelligence and anxiety, stress, and depression in first phase medical undergraduates. Asian J Med Sci.

[6] Downey LA, Johnston JP, Hansen K, Schembri R, Stough C, Tuckwell V, Schweitzer I (2008). The relationship between emotional intelligence and depression in a clinical sample. Eur J Psyc.

[7] Brackett MA, Rivers SE, Shiffman S, Lerner N, Salovey P (2006). Relating emotional abilities to social functioning: a comparison of self-report and performance measures of emotional intelligence. J Pers Soc Psychol.

[8] Cumberland-Li A, Eisenberg N, Champion C, Gershoff E, Fabes RA (2003). The relation of parental emotionality and related dispositional traits to parental expression of emotion and children's social functioning. Motiv Emot.

[9] Zhou Q, Eisenberg N, Losoya SH (2002). The relations of parental warmth and positive expressiveness to children's empathy-related responding and social functioning: a longitudinal study. Child Dev.

[10] Sarkar S, Menon V, Padhy S, Kathiresan P (2024). Mental health and well-being at the workplace. Indian J Psychiatry.

[11] Vogel DL, Heimerdinger-Edwards SR, Hammer JH, Hubbard A (2011). "Boys don't cry": examination of the links between endorsement of masculine norms, self-stigma, and help-seeking attitudes for men from diverse backgrounds. J Couns Psychol.

[12] Salve HR, Jaiswal A, Rath R, Sagar R, Vishnubhatla S (2024). Prevalence and gender difference in depression in primary health care in India: a systematic review and meta-analysis. J Family Med Prim Care.

[13] (2025). Depression Anxiety Stress Scales (DASS). http://www2.psy.unsw.edu.au/dass/.

[14] Henry JD, Crawford JR (2005). The short-form version of the Depression Anxiety Stress Scales (DASS-21): construct validity and normative data in a large non-clinical sample. Br J Clin Psychol.

[15] Schutte NS, Malouff JM, Hall LE, Haggerty DJ, Cooper JT, Golden CJ, Dornheim L (1998). Development and validation of a measure of emotional intelligence. Pers Individ Differ.

[16] Gandhi PA, Kishore J (2020). Prevalence of depression and the associated factors among the software professionals in Delhi: a cross-sectional study. Indian J Public Health.

[17] Arvind BA, Gururaj G, Loganathan S (2019). Prevalence and socioeconomic impact of depressive disorders in India: multisite population-based cross-sectional study. BMJ Open.

[18] Deswal BS, Pawar A (2012). An epidemiological study of mental disorders at Pune, Maharashtra. Indian J Community Med.

[19] Rao TS, Darshan MS, Tandon A (2014). Suttur study: an epidemiological study of psychiatric disorders in south Indian rural population. Indian J Psychiatry.

[20] Manjunatha N, Jayasankar P, Suhas S, Rao GN, Gopalkrishna G, Varghese M, Benegal V (2022). Prevalence and its correlates of anxiety disorders from India's National Mental Health Survey 2016. Indian J Psychiatry.

[21] Reddy VM, Chandrashekar CR (1998). Prevalence of mental and behavioural disorders in India: a meta-analysis. Indian J Psychiatry.

[22] Sahoo S, Khess CR (2010). Prevalence of depression, anxiety, and stress among young male adults in India: a dimensional and categorical diagnoses-based study. J Nerv Ment Dis.

[23] Srinivasan M, Reddy MM, Sarkar S, Menon V (2020). Depression, anxiety, and stress among rural South Indian women - prevalence and correlates: a community-based study. J Neurosci Rural Pract.

[24] Grases G, Colom MA, Sanchis P, Grases F (2023). Relationship of depression with empathy, emotional intelligence, and symptoms of a weakened immune system. Front Psychol.

[25] Schutte NS, Malouff JM, Thorsteinsson EB, Bhullar N, Rooke SE (2007). A meta-analytic investigation of the relationship between emotional intelligence and health. Pers Individ Differ.

[26] Martins A, Ramalho N, Morin E (2010). A comprehensive meta-analysis of the relationship between emotional intelligence and health. Pers Individ Differ.

[27] Baudry AS, Anota A, Mariette C, Bonnetain F, Renaud F, Piessen G, Christophe V (2019). The role of trait emotional intelligence in quality of life, anxiety and depression symptoms after surgery for esophageal or gastric cancer: a French national database FREGAT. Psychooncology.

[28] Peña-Sarrionandia A, Mikolajczak M, Gross JJ (2015). Integrating emotion regulation and emotional intelligence traditions: a meta-analysis. Front Psychol.

[29] Singh Y, Sharma R (2012). Relationship between general intelligence, emotional intelligence, stress levels and stress reactivity. Ann Neurosci.

[30] Landa JM, López-Zafra E, Martos MP, Aguilar-Luzón M (2008). The relationship between emotional intelligence, occupational stress and health in nurses: a questionnaire survey. Int J Nurs Stud.

[31] Nikolaou I, Tsaousis I (2002). Emotional intelligence in the workplace: exploring its effects on occupational stress and organizational commitment. Int J Organ Anal.

[32] Molnar BE, Buka SL, Kessler RC (2001). Child sexual abuse and subsequent psychopathology: results from the National Comorbidity Survey. Am J Public Health.

[33] Kisely S, Abajobir AA, Mills R, Strathearn L, Clavarino A, Najman JM (2018). Child maltreatment and mental health problems in adulthood: birth cohort study. Br J Psychiatry.

[34] Gottfredson RK, Becker WJ (2023). How past trauma impacts emotional intelligence: examining the connection. Front Psychol.

[35] Grey I, Arora T, Thomas J, Saneh A, Tohme P, Abi-Habib R (2020). The role of perceived social support on depression and sleep during the COVID-19 pandemic. Psychiatry Res.

[36] Dour HJ, Wiley JF, Roy-Byrne P (2014). Perceived social support mediates anxiety and depressive symptom changes following primary care intervention. Depress Anxiety.

